# Engineering and evaluation of thermostable *Is*PETase variants for PET degradation

**DOI:** 10.1002/elsc.202100105

**Published:** 2021-11-29

**Authors:** Stefan Brott, Lara Pfaff, Josephine Schuricht, Jan‐Niklas Schwarz, Dominique Böttcher, Christoffel P. S. Badenhorst, Ren Wei, Uwe T. Bornscheuer

**Affiliations:** ^1^ Department of Biotechnology & Enzyme Catalysis University of Greifswald Institute of Biochemistry Greifswald Germany

**Keywords:** PET hydrolysis, PETase, polyethylene terephthalate, protein engineering, thermostability

## Abstract

Polyethylene terephthalate (PET) is a mass‐produced petroleum‐based synthetic polymer. Enzymatic PET degradation using, for example, *Ideonella sakaiensis* PETase (*Is*PETase) can be a more environmentally friendly and energy‐saving alternative to the chemical recycling of PET. However, *Is*PETase is a mesophilic enzyme with an optimal reaction temperature lower than the glass transition temperature (*T*_g_) of PET, where the amorphous polymers can be readily accessed for enzymatic breakdown. In this study, we used error‐prone PCR to generate a mutant library based on a thermostable triple mutant (TM) of *Is*PETase. The library was screened against the commercially available polyester‐polyurethane Impranil DLN W 50 for more thermostable *Is*PETase variants, yielding four variants with higher melting points. The most promising *Is*PETaseTM^K95N/F201I^ variant had a 5.0°C higher melting point than *Is*PETaseTM. Although this variant showed a slightly lower activity on PET at lower incubation temperatures, its increased thermostability makes it a more active PET hydrolase at higher reaction temperatures up to 60°C. Several other variants were compared and combined with selected previously published *Is*PETase mutants in terms of thermostability and hydrolytic activity against PET nanoparticles and amorphous PET films. Our findings indicate that thermostability is one of the most important characteristics of an effective PET hydrolase.

**Abbreviations**
BHET: bis‐(2‐hydroxyethyl) terephthalate
EG: ethylene glycol
*Is*PETase: PETase from *Ideonella sakaiensis*
LCC: leaf‐branch compost cutinase
MHET: mono‐(2‐hydroxyethyl) terephthalate
PET: polyethylene terephthalate
TA: terephthalic acid
*T*_m_
: melting point
TM: triple mutant

## 1 INTRODUCTION

The combination of mass production of single‐use plastic products, plastic longevity, and insufficient waste management has resulted in an accumulation of plastic in the environment over the last few decades [1]. Globally, approximately 368 million tons of plastic were produced in 2019 alone [2]. Polyethylene terephthalate (PET), a semi‐crystalline thermoplastic composed of the two monomeric units terephthalic acid (TA) and ethylene glycol (EG), linked by ester bonds, is one of the mass‐produced petroleum‐based synthetic polymers [3, 4]. More than 50 million tons of PET are annually produced globally, with the main applications being synthetic fibers in the textile industry and beverage bottles in the packaging sector [5, 6].

Recycling is a viable disposal solution for existing plastic waste in terms of energy savings, material efficiency and circularity, for which the use of enzymes can be an alternative option [7]. PET has hydrolysable ester backbones and is thus more susceptible to enzymatic degradation than petrochemical vinyl plastics with solely carbon‐carbon backbones [8, 9]. Several enzymes capable of cleaving PET have been discovered in recent years [10]. These PET hydrolases belong to the enzyme classes of carboxylesterases [11], lipases [12] and cutinases [13, 14, 15, 16]. When compared to chemical recycling methods, the use of enzymes provides an environmentally friendly and energy‐saving alternative that also reduces the use of hazardous chemicals [7]. Although the industrially‐relevant use of enzymes in PET degradation is still in its early stages, an engineered variant of the leaf‐branch compost cutinase (LCC) has already enabled nearly complete depolymerization of pre‐treated PET bottle waste and virgin polymers were re‐synthesized using the recovered TA [17]. A significant milestone in PET breakdown was the identification of the Gram‐negative aerobic β‐proteobacterium *Ideonella sakaiensis* by screening of 250 environmental samples obtained from a PET bottle recycling site in Japan [18]. The bacterium utilized PET as its main energy and carbon source, and it was able to grow on low‐crystallinity PET film and degrade it almost completely after 6 weeks at an incubation temperature of 30°C under controlled laboratory conditions [18]. Two enzymes, designated as *Is*PETase and *Is*MHETase, are involved in the breakdown of PET by *Ideonella sakaiensis* [18]. *Is*PETase catalyzes the depolymerization of PET to the main product mono‐(2‐hydroxyethyl) terephthalate (MHET) and the side products bis‐(2‐hydroxyethyl) terephthalate (BHET), TA, and EG [18]. *Is*MHETase then hydrolyzes MHET to TA and EG [18].

**PRACTICAL APPLICATION**

In this work, an error‐prone PCR‐generated mutant library was screened using an agar plate assay based on the commercially available polyester‐polyurethane Impranil DLN W 50. With this type of screening, finding multiple thermostable polyester hydrolase variants within a mutant library was possible. Recycling plastics such as PET with enzymes is one of the recently developed methods to mitigate the plastic pollution and to promote the circularity of material flow for a sustainable plastic economy. The enzymatic degradation of polyethylene terephthalate is more feasible at higher incubation temperatures. Therefore, mesophilic enzymes such as the *Is*PETase from *Ideonella sakaiensis* need to be engineered, revealing higher thermostability and activity by protein engineering methods such as directed evolution.

In recent years, the crystal structures of *Is*PETase and *Is*MHETase have been elucidated and published [19, 20, 21, 22, 23, 24, 25]. Despite this, only one *Is*PETase structure has been solved so far with a co‐crystallized ligand analogous to MHET [19]. *Is*PETase exhibits a typical α/β‐hydrolase fold consisting of seven α‐helices and nine β‐strands forming a central twisted β‐sheet [21], which is highly conserved among many other cutinase‐like PET‐hydrolyzing enzymes [26]. The catalytic triad of *Is*PETase, located on the protein surface, is composed of the amino acids S160‐D206‐H237 [22]. The enzyme has two intramolecular disulfide bridges, whereas the structurally homologous cutinases TfCut2 from *Thermobifida fusca* and LCC only have one [19, 27]. The strictly conserved disulfide bridge (C273‐C289) connects the last loop and the C‐terminal helix [19]. The *Is*PETase‐specific disulfide bridge (C203‐C239) is located near the active site and connects two loops containing the residues of the catalytic triad [19]. This disulfide bridge is thought to be responsible for the high room temperature flexibility of the *Is*PETase active site and the resulting high activity against PET [27]. Methods such as structure‐based sequence alignment of *Is*PETase with structural homologues or molecular docking experiments followed by biochemical characterization have also contributed to the elucidation of important residues in the enzyme [19, 20, 23, 24]. For example, a tryptophan residue crucial for substrate binding was discovered in *Is*PETase [19]. This amino acid W185 is located near the catalytic center and can adopt three different conformations, which is why it is also referred as a wobbling tryptophan [19, 23]. In a proposed substrate binding, W185 forms π‐π‐stacking interactions with the phenylene units of PET, allowing interactions with the substrate and facilitating binding [ 19, 23]. The wobbling of W185 in *Is*PETase is based on a serine at position 214 and an isoleucine at 218, which allow the tryptophan to rotate [28]. In homologous enzymes, these serine and isoleucine residues are replaced by histidine or phenylalanine, respectively, restricting the movement of the tryptophan and thus lowering the activity for PET degradation [28].

Unlike LCC or TfCut2 from *T. fusca*, *Is*PETase is a mesophilic enzyme with a melting point (*T*_m_) of approximately 45°C, and it is thus thermally unstable for applications at higher temperatures [16, 29, 30]. The crystallinity of PET influences the efficiency of enzymatic PET degradation [16, 31]. The presence of highly ordered crystalline content in a specific PET sample reduces the overall mobility of the polymer chains and their accessibility to enzymatic hydrolysis [29, 32]. In comparison, the less‐ordered amorphous fraction is significantly more susceptible to enzymatic attack at a reaction temperature close to the glass transition temperature (*T*_g_) of PET [29]. Because enzymatic PET hydrolysis is carried out in aqueous environments, the *T*_g_ of PET is notably reduced to below 60°C due to the plasticization effect of water [33, 34]. As a result, increasing the thermal stability of a desired PET hydrolase for use at temperatures above 60°C has proven to be useful for making better PET‐degrading enzymes [26].

In order to achieve effective PET degradation by *Is*PETase, the activity and thermostability of the enzyme has been improved by protein engineering in previous studies [19, 20, 22–24]. For instance, an improvement of *Is*PETase activity was achieved by the R280A substitution [20]. Docking studies showed that the polar arginine residue in the binding pocket of *Is*PETase hinders stable binding of PET, therefore the substitution of the arginine residue by small hydrophobic amino acid residues leads to increased activity [20]. Son et al. generated an *Is*PETase triple mutant (TM = *Is*PETase^S121E/D186H/R280A^) by combining the R280A mutation with the β6‐β7‐connecting loop‐stabilizing mutations S121E and D186H. The resulting triple mutant exhibited a 14‐fold improved PET hydrolysis activity and its *T*_m_ was increased by 8.81°C [21]. The addition of another disulfide bridge to this triple mutant via N233C and S282C substitutions resulted in a *T*_m_ of 69.4°C and a further 5 to 7‐fold increase in activity [35]. The equivalent disulfide bond has been shown to have similar thermostabilizing and activating effects with the homologous PET hydrolyzing enzymes LCC [17] and TfCut2 [36]. Cui et al. used a computer‐assisted strategy called GRAPE (greedy accumulated strategy for protein engineering) to create another *Is*PETase variant [37]. This variant (*Is*PETase^L117F/Q119Y/T140D/W159H/G165A/I168R/A180I/S188Q/S214H/^ ^R280A^) was named *Dura*PETase because its *T*_m_ was increased by 31°C and it had increased degradation activity against highly crystalline PET film [37].

In this study, we used error‐prone PCR to generate a mutant library based on the triple mutant of *Is*PETase [21]. The library was screened for thermostable *Is*PETase variants against the commercially available polyester‐polyurethane Impranil DLN W 50. Selected variants were then combined with other previously published promising *Is*PETase mutants (Table 1) and investigated in terms of thermostability and hydrolytic activity against PET nanoparticles and amorphous PET films.

**TABLE 1:** Overview of previously published promising *Is*PETase variants which have served as templates for this study

*Is*PETase variant	Amino acid substitutions	References
*Is*PETaseTM	*Is*PETase^S121E/D186H/R280A^	[21]
*Is*PETaseTM^N233C/S282C^	*Is*PETase^S121E/D186H/N233C/R280A/S282C^	[35]
*Dura*PETase	*Is*PETase^L117F/Q119Y/T140D/W159H/G165A/I168R/A180I/S188Q/S214H/R280A^	[37]

## 2 MATERIALS AND METHODS

Chemicals and consumables were purchased from Sigma Aldrich (Steinheim, Germany), Carl Roth (Karlsruhe, Germany), Fermentas (St. Leon‐Rot, Germany), Fluka (Buchs, Switzerland), Thermo Fisher Scientific (Waltham, MA, USA), Merck KGaA (Darmstadt, Germany), ChiroBlock GmbH (Wolfen, Germany) and New England Biolabs GmbH (Frankfurt am Main, Germany). Oligonucleotide primers (Table S1) were ordered from Thermo Fisher Scientific (Waltham, MA, USA). The polyester‐polyurethane emulsion Impranil DLN W 50 was a kind gift provided by CSC JÄKLECHEMIE GmbH & Co. (Hamburg, Germany).

### 2.1 Generation of *Is*PETase variants

Synthetic genes, codon optimized for expression in *Escherichia coli*, encoding the wild‐type *Is*PETase from *I. sakaiensis*, *Dura*PETase, and *Dura*PETase^K95N/S121E/D186H/F201I^, were synthesized and cloned into the pET‐21b vector by BioCat GmbH (Heidelberg, Germany). The constructs encoded the recombinant proteins as fusions to C‐terminal His_6_‐tags for affinity purification, for purification method see Supporting Information. The TM was generated based on the wild‐type gene using the Q5 Site‐Directed Mutagenesis Kit (New England Biolabs GmbH, Frankfurt am Main, Germany). For the generation of the mutant library based on the triple mutant, the GeneMorph II Random Mutagenesis Kit (Agilent Technologies Inc., Santa Clara, CA, USA) was used. The error‐prone PCR amplicon was cleaned up using the NucleoSpin Gel and PCR Clean‐up kit (MACHEREY‐NAGEL GmbH & Co. KG, Düren, Germany) and used as a MegaPrimer for MEGAWHOP cloning [38] using *Pfu*Plus! DNA Polymerase (EURx, Gdansk, Poland). To remove the template plasmid from the library, the MEGAWHOP PCR product was digested with DpnI (New England Biolabs GmbH, Frankfurt am Main, Germany). The library was transformed into electrocompetent *E. coli* TOP10 (Thermo Fisher Scientific, Waltham, MA, USA) and 0.1% of it was sequenced to investigate the mutation spectrum. Sanger Sequencing was performed using Mix2Seq Kits from Eurofins Genomics Germany GmbH (Ebersberg, Germany). Substitutions of K95N/F201I and/or N233C/S282C in wild‐type *Is*PETase, *Dura*PETase, and *Dura*PETase^K95N/S121E/F201/R280A^ were introduced by QuikChange using *Pfu*Plus! DNA Polymerase, followed by DpnI digestion and transformation into chemically competent *E. coli* TOP10. The expression of active *Is*PETase variants is described in the Supporting Information.

### 2.2 Screening based on Impranil agar plates

Impranil DLN W 50 agar plates were used for the screening of the *Is*PETaseTM mutant library. The lysogeny broth (LB) agar plates contained 0.5 mM isopropyl‐β‐d‐thiogalactopyranoside (IPTG), 100 μg mL^−1^ ampicillin, and 0.5% Impranil DLN W 50, diluted from a 40% suspension. The mutant library was used to transform chemically competent *E. coli* SHuffle T7 Express (New England Biolabs GmbH, Frankfurt am Main, Germany) cells which were then spread onto LB agar plates containing 100 μg mL^−1^ ampicillin. After the cells were incubated overnight at 30°C, colonies were picked and transferred to another LB‐ampicillin agar plate (storage plate) and in parallel to the Impranil agar plate (assay plate). The storage and assay plates were first incubated overnight at 30°C, the assay plates were subsequently incubated at 60°C for 24 h. Degradation of Impranil leads to formation of clear zones around colonies expressing active *Is*PETase variants. An increased size of the haloes formed, relative to the TM as control, was used as a preliminary indication of increased thermostability. Selected colonies were picked from the storage plate and used to inoculate an overnight culture for plasmid isolation using the innuPREP Plasmid Mini Kit (Analytik Jena GmbH, Jena, Germany). Increased thermostability was then experimentally verified by protein expression, purification and nanoDSF, as described below.

### 2.3 Determination of protein concentration

Protein concentrations were determined using the Pierce BCA Protein Assay Kit (Thermo Fisher Scientific, Waltham, MA, USA).

### 2.4 Measurement of melting points

Determination of the *T*_m_ for each *Is*PETase variant was performed by nanoDSF using the Prometheus NT.48 (NanoTemper Technologies, Munich, Germany). The measurement was performed in 50 mM sodium phosphate (pH 7.5) using a protein concentration of 0.5 mg mL^−1^. Temperature was scanned at 1°C per minute between 20°C and 95°C. The instrument has a fixed excitation wavelength of 285 nm in combination with emission wavelengths of 330 and 350 nm.

### 2.5 Degradation of PET nanoparticles and PET film

PET nanoparticles were prepared based on previous publications [39, 40]. The degradation of PET nanoparticles (0.2 mg mL^−1^) was performed with an *Is*PETase concentration of 30 nM in 200 μL of 50 mM sodium phosphate buffer (pH 7.5). Enzymatic hydrolysis was performed at different incubation temperatures ranging from 30°C to 60°C and a constant agitation of 1000 rpm for 24 h. For the biocatalysis with amorphous PET film (Goodfellow GmbH, Bad Nauheim, Germany), the film was cut into 1 × 2 cm^2^ pieces (∼60 mg) and washed with a solution containing sodium dodecyl sulfate (SDS), distilled water, and ethanol. The PET film was then dried at 50°C for 24 h. The degradation of PET film was performed in 50 mM glycine‐NaOH buffer (pH 9.0) with an *Is*PETase concentration of 50 nM in a reaction volume of 1.5 mL at 60°C and constant agitation of 1000 rpm for 72 h. To quench the reaction, PET nanoparticles were first removed by centrifugation at 17,000 × *g* for 10 min at 4°C. Next, 100 μL of the supernatant was added to an equal volume of 200 mM sodium phosphate (pH 2.5) containing 20% v/v dimethyl sulfoxide (DMSO). This mixture was then incubated at 95°C for 10 min. To quench the reaction with amorphous PET film, the PET film was removed. Then, the same quenching protocol as for PET nanoparticles, without the centrifugation step, was performed. Samples were stored at ‐20°C until measurement by high‐performance liquid chromatography with the Hitachi LaChrom Elite HPLC System (Hitachi, Chiyoda, Japan). Analysis of MHET, BHET, and TA by HPLC was performed according to Palm et al. [25]. Briefly, PET degradation products were analyzed on a Kinetex 5 μm EVO C18 100 Å, 150 × 4.6 mm column (Phenomenex, Aschaffenburg, Germany) with a gradient of acetonitrile and 0.1% v/v formic acid in water at 30°C. Ten microliters of sample was injected and the flow rate was 0.8 mL min^−1^. Acetonitrile was increased from 5% to 44% over 12 min and then to 70% over 15 min, after which the ratio remained constant at 70% acetonitrile for 3 min. MHET, BHET, and TA were detected at 240 nm, and quantification was performed based on calibration curves.

## 3 RESULTS

### 3.1 Screening for thermostable *Is*PETase variants

**FIGURE 1:**
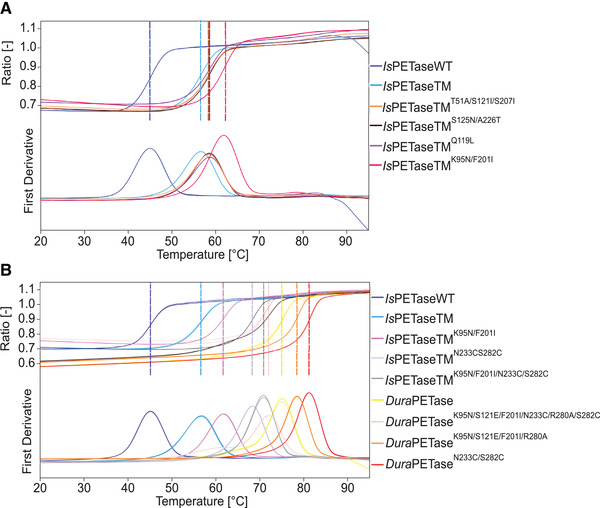
Melting point determination of selected *Is*PETase variants by nanoDSF. (A) Discovered *Is*PETase variants from the screening and (B) constructed *Is*PETase variants by combining different mutations with previously published amino acid substitutions (B). The *T*_m_ can be determined from the position of the curve maximum. The measurement was performed in 50 mM sodium phosphate buffer (pH 7.5) with purified enzymes (0.5 mg mL^‐1^). The measurements were performed in duplicates

Approximately 49,000 clones were screened on Impranil agar plates, accounting for approximately ∼52% of the error‐prone PCR‐based mutant library with ∼95,000 clones. *Is*PETase activity was assessed by observing the halo formation around the bacterial colony. At higher incubation temperatures (≥60°C), the size of the clear zones was thought be dependent on the thermostability of the recombinant variants. Four colonies with significantly increased halo sizes, compared to that of the TM, were selected for further experiments. These were TM^K95N/F201I^, TM^S125N/A226T^, TM^Q119L^ and TM^T51A/S125I/S207I^ (Figure S1). Based on structural analysis of *Is*PETase (Figure S2), six substitutions were present on the surface of the TM. In contrast, the F201I mutation was located deeply inside the enzyme. After expression and purification (Figure S3), the *T*_m_ of these four variants as well as the wild‐type *Is*PETase (**WT**) and TM were determined by nanoDSF (Figure 1A). For WT and TM, *T*_m_ of 45.1°C and 56.6°C, respectively, were measured. Most of the variants displayed an improvement in *T*_m_ of approximately 2°C compared to TM (Table S2). The most thermostable variant was *Is*PETaseTM^K95N/F201I^ (**TM1**) with an increased *T*_m_ of 61.7°C which is 5.3°C higher than that of *Is*PETaseTM.

### 3.2 Engineering of more thermostable *Is*PETase variants

With the aim of generating further more thermostable variants, the most promising stabilizing K95N and F201I substitutions were incorporated into previously published *Is*PETase variants including *Is*PETaseTM^N233C/S282C^ (**TM2**) and *Dura*PETase (**D**). For *Dura*PETase variants, the S121E and R280A substitutions were also investigated simultaneously, since these substitutions in combination with the K95N and F201I substitution led to the high *T*_m_ in the discovered TM1 variant. At the same time the influence of the N233C and S282C substitutions, which were previously described by Zhong‐Johnson et al. [35] exclusively for the *Is*PETaseTM, were also investigated with *Dura*PETase. After expression and purification, the *T*_m_ was determined via nanoDSF for selected *Is*PETase variants (Figure 1B). An increase in *T*_m_ of 2.6°C from 68.2°C to 70.9°C was observed for the *Is*PETaseTM^K95N/F201I/N233C/S282C^ (**TM3**) variant (Table 2). Introduction of the substitutions K95N, S121E, R280A and F201I led to a decrease of the *T*_m_ by 3°C from 75.0°C to 71.9°C in *Dura*PETase^K95N/S121E/F201I/R280A^ (**D2**). In comparison, by introducing the double cysteine residues, the *T*_m_ of *Dura*PETase variants were further increased, to 81.1°C for *Dura*PETase^N233C/S282C^ (**D1**) and 78.4°C for *Dura*PETase^K95N/S121E/F201I/N233C/R280A/S282C^ (**D3**). Consequently, *T*_m_ has been increased by 36.1°C from 45.1°C to 81.1°C for D1 compared to the WT by incorporating the previously reported N233C and S282C substitutions.

**TABLE 2:** Melting points of selected *Is*PETase variants, which were generated by combining the K96N/F201I substitutions with other previously described *Is*PETase mutants

	*Is*PETase variant	Melting point ± SD [°C]	*T*_m_ [°C] increase compared to	
			*Is*PETaseWT	*Is*PETaseTM
WT	*Is*PETaseWT	45.1 ± 0.1	–	–
TM	*Is*PETaseTM	56.6 ± 1.6	11.5	–
TM1	*Is*PETaseTM^K95N/F201I^	61.6 ± 0.1	16.6	5.1
TM2	*Is*PETaseTM^N233C/S282C^	68.2 ± 0.1	23.2	11.6
TM3	*Is*PETaseTM^K95N/F201I/N233C/S282C^	70.8 ± 0.1	25.8	14.3
D	*Dura*PETase	75.0 ± 0.1	29.9	18.4
D1	*Dura*PETase^N233C/S282C^	81.1 ± 0.1	36.1	24.6
D2	*Dura*PETase^K95N/S121E/F201I/R280A^	71.9 ± 0.1	26.9	15.3
D3	*Dura*PETase^K95N/S121E/F201I/N233C/R280A/S282C^	78.4 ± 0.1	33.3	21.8

Data were determined by nanoDSF with purified enzymes (0.5 mg mL^−1^) in 50 mM sodium phosphate buffer (pH 7.5). The measurements were performed in duplicates. The mean values and the standard deviations (SD) are given.

### 3.3 Influence of substitutions on PET nanoparticle and amorphous PET film hydrolysis

**FIGURE 2:**
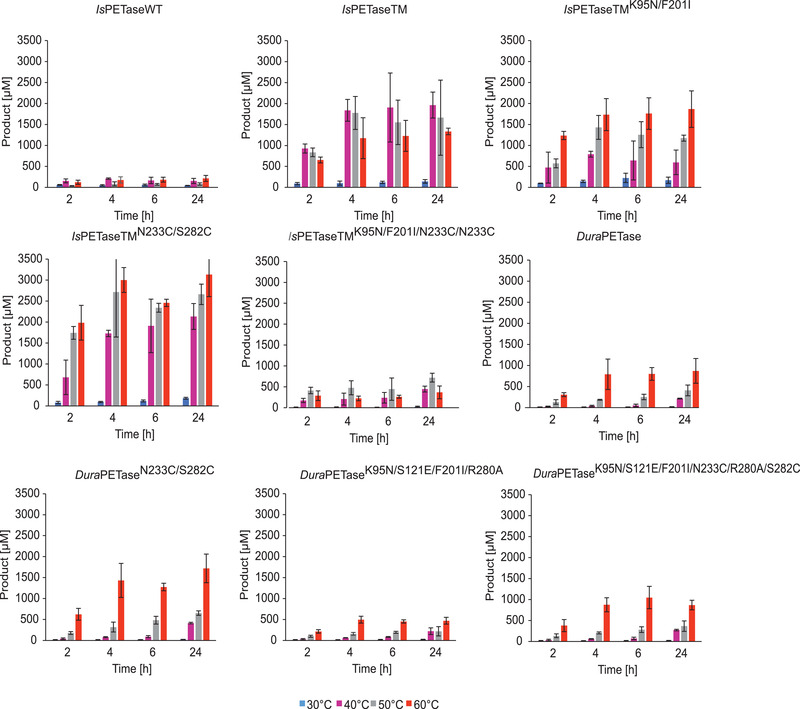
Time course of total product release at different incubation temperatures. The total product released refers to the sum of released MHET, TA, and BHET. Biocatalysis with PET nanoparticles was performed with 30 nM *Is*PETase variant in 50 mM sodium phosphate buffer (pH 7.5) at the respective incubation temperatures and a constant agitation of 1000 rpm for 24 h. A final PET nanoparticle concentration of 0.2 mg mL^−1^ was used. The measurements were performed in triplicates and the mean values and standard deviations are given

**FIGURE 3:**
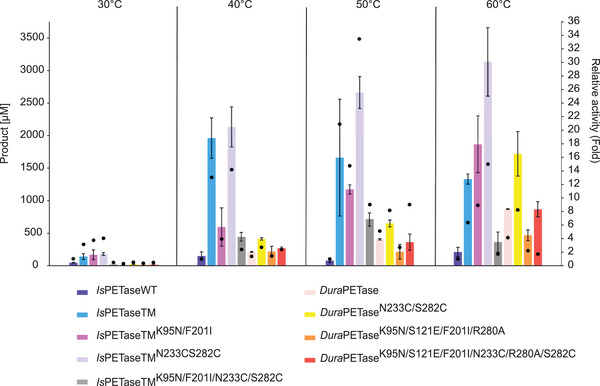
Total product release after degradation of PET nanoparticles using selected *Is*PETase variants after 24 h and an incubation temperature of 60°C. Black dots represent the relative activity compared to wild‐type *Is*PETase. Biocatalysis with PET nanoparticles was performed with 30 nM *Is*PETase variant in 50 mM sodium phosphate buffer (pH 7.5) at the incubation temperature of 60°C and a constant agitation of 1000 rpm for 24 h. A final PET nanoparticle concentration of 0.2 mg mL^−1^ was used. The total product released refers to the sum of released MHET, TA, and BHET. The measurements were performed in triplicates and the mean values and standard deviations are given

The hydrolysis of PET nanoparticles with aforementioned *Is*PETase variants was investigated at different temperatures ranging from 30°C to 60°C for 24 h. With increasing temperature, an increased degradation rate of PET nanoparticles in terms of higher product release could be observed for each variant (Figure 2). A notably improved performance in PET hydrolysis was observed with all variants when the incubation temperature was increased from 30°C to 40°C. Even the WT showed increased activity at higher temperatures, although the *T*_m_ is only 45°C (Table 2) and the overall yield of degradation products was markedly lower than with the other variants. Comparing the TM related variants, it can be observed that the introduction of the K95N and F201I substitution into the existing TM2 variant as well as into the TM resulted in a decrease in total product release, defined as the sum of released MHET, TA, and BHET, for PET nanoparticle hydrolysis (Figure 3). The relative activity compared to the TM decreased by approximately 70% for TM1 when incubated at 40°C (Table S3). Whereas at 50°C a reduction in the relative activity of only 30% was observed. However, it was also shown that at 60°C, more product was released from the TM1 variant compared to the TM. Comparable relative activities were observed at 60°C for TM and TM1. At this incubation temperature, an 8‐fold enhancement was observed for TM1 in relative activity compared with wild‐type *Is*PETase (Figure 3) making TM1 possessing the second largest increase after TM2. Most product formation was observed at 60°C with the TM2 variant (Figure 3). For the combination of K95N/F201I and N233C/S282C substitutions (TM4), a strongly reduced relative activity (‐70%) compared to TM at 60°C (Table S3) and a low level of total product release were observed. The K95N/S121E/F201I/R280A substitutions in the *Dura*PETase (D2) also resulted in a reduced product release during the hydrolysis of PET nanoparticles compared to other *Dura*PETase variants at 60°C. However, the degradation of PET nanoparticles was improved with the *Dura*PETase variants containing the N233C/S282C mutations. A two‐fold increase in relative activity was observed as a result of this substitution for D1 at both 50°C and 60°C (Figure 3). In particular, for the *Dura*PETase variants, increased degradation of PET was shown at 60°C compared to the other incubation temperatures (Figure 2). The main product formed during the hydrolysis of PET nanoparticles was MHET (Figure S4). TA and very low BHET concentrations were also detected.

**FIGURE 4:**
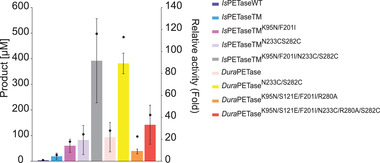
Total product release after degradation of amorphous PET film for selected *Is*PETase variants after 72 h at an incubation temperature of 60°C. Black dots represent the relative activity compared to wild‐type *Is*PETase. For biocatalysis with amorphous PET film, an enzyme concentration of 50 nM was used. The reaction was carried out in 50 mM glycine‐NaOH buffer (pH 9.0). The PET film was incubated at 60°C and a constant agitation of 1000 rpm for 3 days. The total product released refers to the sum of released MHET, TA, and BHET. The measurements were performed in triplicates and the mean values and standard deviations are given

**FIGURE 5:**
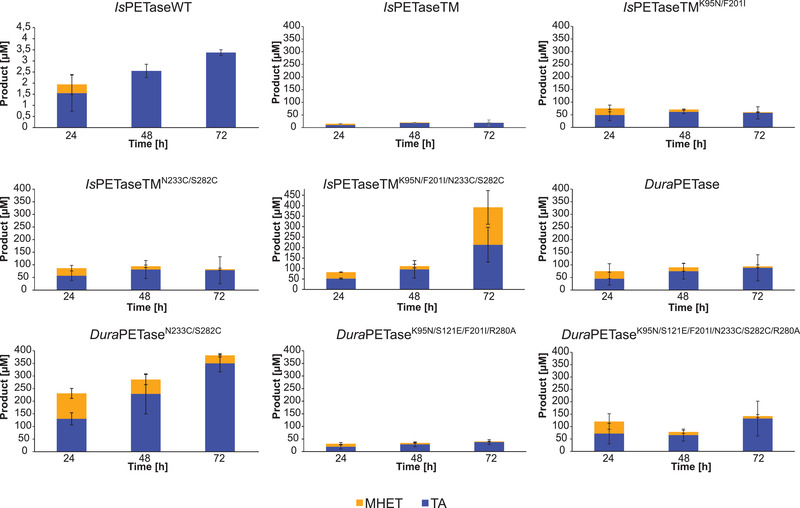
Comparison of degradation products for amorphous PET film hydrolysis catalyzed by selected *Is*PETase variants over the time course of 72 h at an incubation temperature of 60°C. Biocatalysis with amorphous PET film was performed with 50 nM of each *Is*PETase variant in glycine‐NaOH buffer (pH 9.0). The PET film was incubated at 60°C with constant agitation at 1000 rpm for 3 days. The measurement was performed in triplicates and the mean values and standard deviations are given

The degradation of amorphous PET film was carried out at an incubation temperature of 60°C for 72 h. Under these conditions, almost no product formation was observed for WT. Except for D2, almost all thermostable variants led to higher total product concentration when compared to unstable variants like TM (Figure 4). In addition, higher relative activities than TM were observed for all variants except for WT and D2 (Table S4). Surprisingly, compared to the degradation performance obtained with PET nanoparticles, the combination variant (TM3) exhibited one of the highest relative activities compared to WT (120‐fold increase, Figure 4) and TM (10 to 20‐fold increase, Table S4). The highest total product was yielded with D1 and the TM3 variant. However, unlike the hydrolysis of PET nanoparticles, the main product this time was TA rather than MHET (Figure 5).

## 4 DISCUSSION

Screening methods based on agar plate assays containing PET or polycaprolactone nanoparticles have been developed to identify polyesterases from metagenomic libraries [41]. However, these nanoparticles can precipitate during agar plate preparation, interfering with uniform distribution in the agar. Therefore, a suspension of the aliphatic polyester‐polyurethane Impranil DLN W 50 can be applied instead [41]. The formation of clear zones on the agar plate around bacterial colonies indicates the functional expression of catalytically active polyester hydrolases.

All *Is*PETase variants discovered here with the Impranil agar plate screening showed an increased *T*_m_ compared the wild‐type enzyme, thereby validating the criterium based on halo size. An improvement of 16.9°C compared to the wild‐type was observed for the *Is*PETaseTM^K95N/F201I^ variant. Thermostability strongly depends on the flexibility of the protein. A variety of interactions, such as ionic interactions, disulfide bonds, hydrogen bonds, and hydrophobic interactions, affect the flexibility and thus the thermostability of the protein [42]. An increase in stability can be mediated either by stabilization of the folded form or by destabilization of the unfolded form [43]. The substitution of an amino acid must thereby affect the folded and unfolded forms differently [43]. Finding an explanation for increased thermostability is therefore usually very difficult.

For the WT and the TM, *T*_m_ of 45.1°C and 56.6°C, respectively, could be measured by nanoDSF. The *T*_m_ determined with nanoDSF have small deviations in the range of about 2°C compared to the reported *T*_m_ for WT, TM, TM2 and D. For instance, in this work a *T*_m_ increase of 29.9°C was observed for the *Dura*PETase, whereas in the publication by Cui et al. [37] an increase of 31°C was mentioned. As summarized in Table S5, the methods and exact equipment used for the *T*_m_ measurement in different studies may be responsible for these small discrepancies.

The combination of the S125N and A226T substitutions showed an increase in thermostability in this study. However, the computationally predicted single mutations S125R and A226P for WT did not cause an increase or even a decrease in *T*_m_ as reported by Cui et al. [37]. The impact of a substitution is highly dependent on the type and number of mutations already present in an enzyme [44]. This could also be the reason for the lack of an increase and the decrease in *T*_m_ observed for the *Dura*PETase variants, which already contain the K95N/S121E/F201I and R280A substitutions. The K95 position was already evaluated in the publication reporting *Dura*PETase, where a K95A mutation only led to an increase of 2.5°C in *T*_m_ for the WT [37]. The K95N substitution could have the same influence on the thermostability as the K95A substitution, additional increase in thermostability would possibly result from the F201I substitution.

The introduction of additional disulfide bridges into an enzyme is one possible strategy to increase thermostability [43]. The main goal of these cross‐links is to reduce the flexibility of certain thermolabile folds [43]. Crosslinking results in a reduced entropic state during the unfolding process, shifting the equilibrium toward the folded form [43]. The selection of the correct positions for amino acid substitution is crucial, the substitution should have no effect on the catalytic properties of the enzyme. For the identification of suitable positions for disulfide bridges in PET degrading enzymes, the structures of homologous cutinases can be helpful [17, 45]. For Cut190 from *Saccharomonospora viridis*, which is homologous to *Is*PETase [35], the *T*_m_ could be increased by more than 20°C through replacing calcium‐binding sites with disulfide bridges [45]. This led, for instance, to the discovery of the N233C and S282C substitutions in *Is*PETase [35]. However, since no crystal structure was described for the *Is*PETase^N233C/S282C^ variant, it can only be speculated on the formation of the disulfide bridge in the *Is*PETase variants. In this study the insertion of the N233C and S282C substitutions into the *Dura*PETase also led to an increase in *T*_m_ of approximately 6.1°C. This D1 variant revealed indeed the highest *T*_m_ of 81.1°C among the so far described *Is*PETase variants.

At higher incubation temperatures, a higher product release was observed in most cases for the thermostabilized *Is*PETase variants. As the flexibility of the amorphous polymer chains will significantly increase at temperatures close to the *T*_g_ of PET, better enzymatic degradation performance can be expected [16, 29]. Increased thermostability is therefore advantageous for the *Is*PETase‐catalyzed degradation of PET. A good example in this study is the comparison of the TM and TM1 variants. In the degradation of PET nanoparticles, the TM variant showed a higher total product formation at 40°C than TM1. This variant also possessed only a relative activity of 30.4% compared to TM at this incubation temperature. Only when the degradation was carried out at 60°C, a similar relative activity was observed between TM and TM1 (Table S3). The influence on PET degradation due to increased thermostability is better observed for the TM1 variant when degrading amorphous PET film. There, this variant showed an approximately two‐fold increase in relative activity compared to TM (Table S4). Since TM only has a T*_m_* of 56.5°C, it is highly probable that this variant is inactivated much faster over the period of 72 h than TM1 and therefore less product was released (Figure 4).

For variants containing the K95N and F201I substitutions, low activities for hydrolysis of PET nanoparticles were observed. The amino acid F201 is located in a deeply hidden hydrophobic core of *Is*PETase, which additionally consists of positions W97, L101, M157, L199, L230, W257 and M258 [37]. Potentially, the F201I substitution has a structural influence on the active site, since D206 is located only five amino acid residues away from this substitution. By contrast, the high thermostability of TM4 (T_m _= 70.8°C) in combination with the prolonged incubation time of 72 h may be responsible for the increased product release in the degradation of amorphous PET film. Only the D1 variant with the *T*_m_ of 81.1°C possessed a similar relative activity to TM4. Both variants support the principle that an increased *T*_m_ brings a substantial advantage for the degradation of PET.

MHET was formed as the main product of PET nanoparticle hydrolysis. This is consistent with the observations by Yoshida et al. who also showed that MHET was the major product for *Is*PETase‐catalyzed hydrolysis of PET [18]. According to Yoshida et al., degradation of MHET by *Is*PETase should not be possible [18]. However, in the degradation of amorphous PET film, TA is the main product, since MHET is degraded by *Is*PETase with extended incubation time. The same reaction has already been described elsewhere [46]. A prolonged incubation of the PET at high temperatures can lead to a slow transformation of mobile amorphous fractions to rigid amorphous fractions, which are less degradable by the enzyme [16]. This process is called physical aging and can therefore be considered as a competitive reaction to the enzymatic degradation of the amorphous PET [16]. Since an amorphous PET film has much less accessible surface area and much longer polymer chains compared to the PET nanoparticles [47], *Is*PETase and its variants, which cannot take part in the effective degradation of the polymers, will be more likely to degrade the MHET. This effect is further enhanced by the prolonged incubation time of 72 h. Only very low concentrations of BHET were observed for each variant in biocatalysis (Figure S4). It is known that *Is*PETase can catalyze the hydrolysis of BHET to TA and EG [18].

Several more thermostable *Is*PETase variants were discovered in this study. Higher thermostability could also be achieved by combination of four stabilizing substitutions with already promising published variants. For the effective degradation of PET by enzymes, the right balance between hydrolytic activity and thermostability is essential. As demonstrated with *Is*PETase variants containing the K95N and F201I substitutions, even a slightly negative influence of a mutation on the activity of PET hydrolysis can be compensated by the simultaneous increase of *T*_m_ for degradation reaction at higher incubation temperatures. This led to an improved effectiveness in PET degradation by these *Is*PETase variants. An increase in thermostability is possibly accompanied by an increase in rigidity [42] which may cause an activity reduction [48]. Specifically, for PET degradation, the enzymatic activity loss may be compensated by the increased polymer substrate accessibility at higher temperatures. Nonetheless, thermostabilized *Is*PETase variants without significant loss of hydrolytic activity will be of greater interest. The same approach of combining substitutions that increase thermostability without significantly affecting PET hydrolysis activity with mutations that increase activity has been also recently verified by Tournier et al. with LCC to be useful for engineering efficient PET hydrolases.

## CONFLICT OF INTEREST

The authors declare no conflict of interest. No experiments involving animals or humans were performed in the context of this study.

## Supporting information



Supporting Information

## Data Availability

Data available on request from the authors.
