# Supplementary material for: Engineering and evaluation of thermostable IsPETase variants for PET degradation
Source: Eng Life Sci. 2021 Nov 29;22(3-4):192–203. doi: 10.1002/elsc.202100105 (PMC8961046; doi:10.1002/elsc.202100105)
Supplement: Supplementary file 1 — Supporting Information [file ELSC-22-192-s001.pdf]

Supporting information

Engineering and evaluation of thermostable *Is*PETase variants for PET degradation

Stefan Brott, Lara Pfaff, Josephine Schuricht, Jan-Niklas Schwarz, Dominique Böttcher, Christoffel P. S. Badenhorst, Ren Wei, Uwe T. Bornscheuer

Department of Biotechnology & Enzyme Catalysis, University of Greifswald Institute of Biochemistry, Greifswald, Germany

Correspondence: Prof. Dr. Uwe T. Bornscheuer (uwe.bornscheuer@uni-greifswald.de). Department of Biotechnology & Enzyme Catalysis, University of Greifswald Institute of Biochemistry, Felix-Hausdorff-Straße 4, 17489 Greifswald, Germany.

Oligonucleotide primers

Table S1: Used Oligonucleotide primers (5´→3') for generation of *Is*PETase variants.

**Oligonucleotide primers for generating *Is*PETaseTM per Q5® Site-Directed Mutagenesis Kit**	
Fw_*Is*PETase_S121E	ggatcagccggagagccgtagcag
Rv_*Is*PETase_S121E	agggtgctgttggtgtca
Fw_*Is*PETase_D186H	agcgccgtggcatagcagcaccaac
Rv_*Is*PETase_D186H	tgcggcgccgccgctttc
Fw_*Is*PETase_R280A	gaatagcaccgcggtgagcgacttcc
Rv_*Is*PETase_R280A	ggattttcgcacgcaaag
**Oligonucleotide primers for error-prone PCR**	
Fw_*Is*PETase_ep	cagaccaatccgtatgcgc
RV_*Is*PETase_ep	ctcgaggctgcagttcgc
**Oligonucleotide primers introducing N233C and S282C substitutions per QuikChange**	
Fw_*Is*PETaseTM_N233C	cagttcctggaaatttgcggtggcagccacag
Rv_*Is*PETaseTM_N233C	ctgtggctgccaccgcaaatttccaggaactg
Fw_*Is*PETaseTM_S282C	gaatagcaccgcggtgtgcgacttccgtac
Rv_*Is*PETaseTM_S282C	gtacggaagtcgcacaccgcggtgctattc
Fw_*Dura*PETase_N233C	caaacagtttctggaaatttgcggcggcagccatagctgc
Rv_*Dura*PETase_N233C	gcagctatggctgccgccgcaaatttccagaaactgtttg
Fw_*Dura*PETase_S282C	gaatagcaccgccgtttgcgattttcgcaccg
Rv_*Dura*PETase_S282C	cggtgcgaaaatcgcaaacggcggtgctattc
Fw_*Dura*PETase$^{K95N/S121E/F201I/R280A}$_N233C	caaaacagtttctggaaatttgcggtggtagccatagctgcg
Rv_*Dura*PETase$^{K95N/S121E/F201I/R280A}$_N233C	cgcagctatggctaccaccgcaaatttccagaaactgttttg
Fw_*Dura*PETase$^{K95N/S121E/F201I/R280A}$_S282C	gaatagcaccgccgtgtgcgattttcgcaccg
Rv_*Dura*PETase$^{K95N/S121E/F201I/R280A}$_S282C	cggtgcgaaaatcgcacacggcggtgctattc

Screening

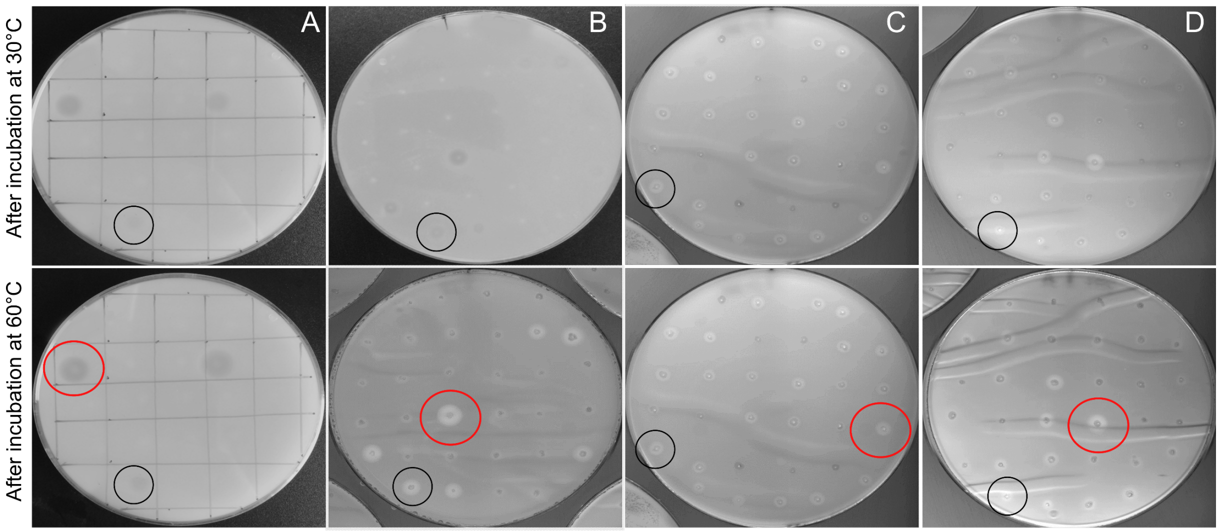

Figure S1: Impranil® agar plates that showed distinguishable haloes in the screening compared to the control. *Is*PETase activity is associated with a halo formation around the cell colony. Colonies expressing possibly more thermostable *Is*PETase variants are circled in red: A) *Is*PETaseTM$^{S125N/A226T}$, B) *Is*PETaseTM$^{K95N/F201I}$, C) *Is*PETaseTM^{Q119L} and D) *Is*PETaseTM$^{T51A/S125I/S207I}$. Each agar plate had at least one colony (outlined in black) expressing *Is*PETaseTM as control. Plates were incubated for 24 hours at 30°C and then for 24 hours at 60°C. Images were colored gray for better visibility.

Positions of discovered amino acid substitution in *Is*PETase$^{S121E/D186H/R280A}$

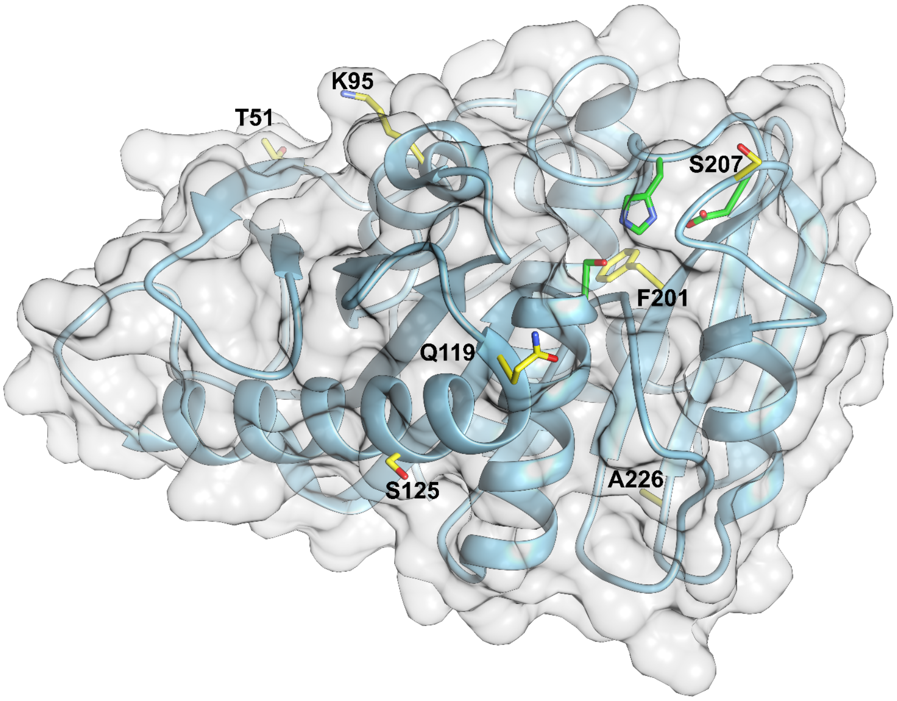

Figure S2: Positions of amino acid substitution within the *Is*PETase triple mutant. This figure shows only the positions of the mutations which were discovered and not the amino acids substitutions. Important amino acids are shown as sticks. The catalytic triad (S160-D206-H237) is colored green and the position of mutation sites is colored yellow. Visualization was done with UCSF Chimera [1]. The structure is based on the crystal structure (Protein Data Bank code 6IJ6) [2]. The crystal structure was not altered.

Expression & Purification

Plasmids of the wild type *Is*PETase and variants based on the triple or quintuple mutants were transformed into chemically competent *E. coli* SHuffle® T7 Express cells (New England Biolabs GmbH, Frankfurt am Main, Germany) and spread onto LB agar plates containing 100 µg mL^{-1} ampicillin. The plates were incubated overnight at 30°C. One colony was picked and used to inoculate an overnight culture containing 100 µg mL^{-1} ampicillin. Cultivation was performed in 400 mL LB medium containing 100 µg mL^{-1} ampicillin in a 1 L flask. The medium was inoculated with overnight culture and incubated at 33°C and 160 rpm until an optical density (OD_{600}) of 1.0 was reached. Expression of the target enzyme was then induced by the addition of 1 mM IPTG. Cultivation was subsequently continued at 16°C and 160 rpm overnight. Cells were harvested by centrifugation at 3,500 *g* and 4°C for 50 min. The cell pellet was washed with sodium phosphate (50 mM, pH 7.5) and subsequently stored at -20°C until cells were lysed. The same protocol was used for expression of *Dura*PETase variants with the exception that the plasmids were transformed into chemically competent *E. coli* OverExpress C43(DE3) cells (Biosearch Technologies, Hoddesdon, United Kingdom). For ultrasonication using a Sonoplus HD 2070 ultrasonic homogenizer (Bandelin electronic GmbH & Co. KG, Berlin, Germany), the cell pellets were resuspended in buffer A (50 mM Tris-HCl buffer, pH 7.5, 150 mM sodium chloride, and 10 mM imidazole). Cell debris was then removed by centrifugation at 10,000 *g* and 4°C for 20 min. Purification was performed with the ÄktaPure chromatography system (GE Healthcare Europe GmbH, Freiburg, Germany) using a HisTrap™ FF crude 5 mL column (Cytiva Europe GmbH, Freiburg, Germany). Undesired proteins were first washed away with buffer A and then with buffer A supplemented with 100 mM imidazole. Elution of *Is*PETase variants was then performed with buffer A supplemented with 200 mM imidazole. Elution fractions containing the protein of interest were pooled and concentrated using Vivaspin 6 centrifugal concentrator (10 kDa MWCO, Sartorius AG, Göttingen, Germany). PD-10 Desalting Columns (GE Healthcare, Freiburg, Germany) were used to exchange the elution buffer to 50 mM sodium phosphate buffer (pH 7.5).

A difference in expression between wild type and mutants is observed. All variants based on *Is*PETaseTM have a better expression than variants based on *Dura*PETase. The differences in expression levels can also be recognized in the SDS-PAGE gels (Figure S3), for example in the lysate fractions. However, after purification, sufficient protein concentrations are present for melting point determinations and biocatalysis.

Sodium dodecyl sulfate–polyacrylamide gel electrophoresis (SDS-PAGE)

Samples were mixed with a 5-fold stock of SDS sample buffer (Tris HCl buffer, pH 6.8, 100 mM, SDS, 4% w/v, glycerol, 20% v/v, β-mercaptoethanol, 2% v/v, EDTA, 25 mM, bromophenol blue, 0.04% w/v) and denatured by incubation at 95˚C for 10 min. For the SDS-PAGE a 12.5% acrylamide gel (separating gel) and a 4.0 % loading gel was used. Electrophoresis was carried out at 200 V. Proteins were stained with Coomassie Blue (PhastGel® Blue R, Sigma Aldrich, Taufkirchen, Germany) and as reference the *Pierce™ Unstained protein molecular weight marker* (Thermo Scientific, Waltham, MA, USA) was used.

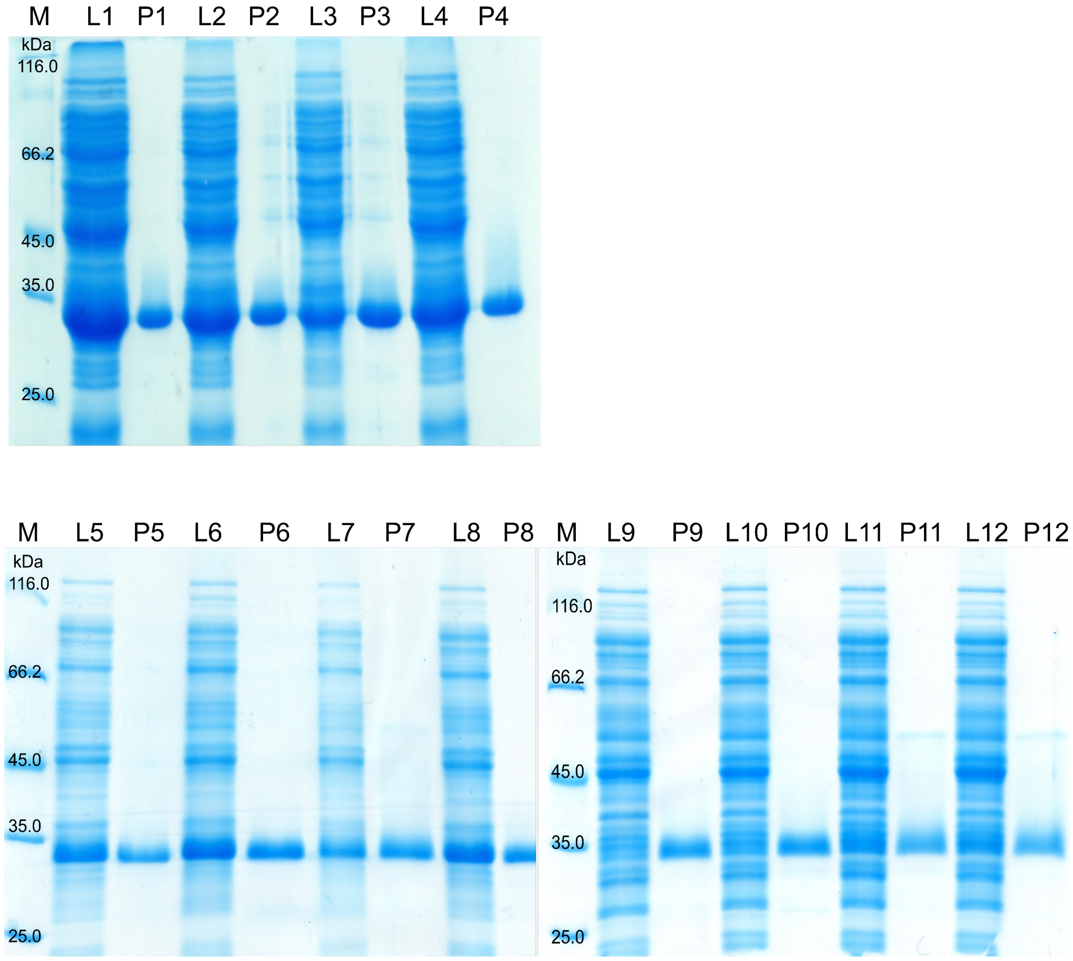

Figure S3: Evaluation of the purity of *Is*PETase variants after purification. Samples of the lysate (L) and the purified pooled fractions (P) were applied. Marker (M) = *Pierce™ Unstained protein molecular weight marker. Is*PETase has a size of approximately 30 kDa. Proteins were stained with Coomassie Blue.

1 = *Is*PETaseTM^{Q119L}

2 = *Is*PETaseTM$^{T51A/S125I/S207I}$

3 = *Is*PETaseTM$^{S125N/A226T}$

4 = *Is*PETaseTM$^{K95N/F201I}$

5 = *Is*PETaseWT

6 = *Is*PETaseTM

7 = *Is*PETaseTM$^{N233C/S282C}$

8 = *Is*PETaseTM$^{K95N/F201I/N233C/S282C}$

9 = *Dura*PETase

10 = *Dura*PETase$^{N233C/S282C}$

11 = *Dura*PETase$^{K95N/S121E/F201I/R280A}$

12 = *Dura*PETase$^{K95N/S121E/F201I/N233C/R280A/S282C}$

Table S2: Melting points of the *Is*PETase variants discovered in the screening with Impranil® agar plates. Data were determined by nanoDSF with purified enzymes (0.5 mg mL^{-1}) in 50 mM sodium phosphate buffer (pH 7.5). The measurement was performed in duplicates and the mean values as well as the standard derivation are given.

**Impranil® agar plate**	***Is*PETase variant**	**Melting point ± SD [°C]**
A)	*Is*PETaseTM$^{S125N/A226T}$	58.5 ± 0.1
B)	*Is*PETaseTM$^{K95N/F201I}$	61.8 ± 0.1
C)	*Is*PETaseTM^{Q119L}	58.6 ± 0.1
D)	*Is*PETaseTM$^{T51A/S125I/S207I}$	58.5 ± 0.1
	*Is*PETaseWT	45.1 ± 0.1
	*Is*PETaseTM	56.6 ± 1.6

Preparation of PET nanoparticles

PET nanoparticles were prepared based on previous publications [3, 4]. Amorphous PET film (300 mg) (Goodfellow GmbH, Bad Nauheim, Germany) was dissolved in 20 mL of 1,1,1,3,3,3-hexafluoro-2-propanol. The dissolved PET was then added dropwise to 300 mL of Milli-Q water under vigorous stirring using an UltraTurrax T25 (IKA Werke GmbH & Co. KG, Staufen im Breisgau, Germany). Solvent was then removed from the mixture by evaporation using a rotary evaporator (Heidolph Instruments GmbH & Co. KG, Schwabach, Germany) and the aqueous suspension further concentrated under vacuum to a final volume of 50 mL. PET nanoparticle concentration was determined gravimetrically.

Table S3: Relative activities of selected *Is*PETase compared to the *Is*PETase triple mutant for degradation of PET nanoparticles at different incubation temperatures. Instead of the wild type the triple mutant was chosen for comparison because it was the starting point for this study. Biocatalysis with PET nanoparticles (0.2 mg mL^{-1}) was performed with 30 nM *Is*PETase variant in 50 mM sodium phosphate buffer (pH 7.5) at different incubation temperatures and constant agitation of 1000 rpm. Relative activities are based on total product release after 24 h incubation. The mean values and standard deviations are given.

	**Relative activity [%] compared to *Is*PETaseTM**			
***Is*PETase variant**	30°C	40°C	50°C	60°C
*Is*PETaseWT	32.3 ± 3.4	7.8 ± 3.1	4.9 ± 1.7	16.0 ± 5.3
*Is*PETaseTM	100.0 ± 30.4	100.0 ± 15.7	100.0 ± 50.4	100.0 ± 5.8
*Is*PETaseTM$^{K95N/F201I}$	119.4 ± 51.5	30.4 ± 14.9	70.6 ± 4.2	139.9 ± 32.7
*Is*PETaseTM$^{N233C/S282C}$	128.4 ± 14.7	108.6 ± 15.7	160.0 ± 14.7	234.7 ± 39.4
*Is*PETaseTM$^{K95N/F201I/N233C/S282C}$	16.5 ± 9.6	18.6 ± 3.6	43.3 ± 6.4	27.5 ± 11.5
*Dura*PETase	10.9 ± 5.6	10.9 ± 0.5	24.6 ± 7.6	65.5 ± 22.0
*Dura*PETase$^{N233C/S282C}$	17.4 ± 1.7	21.1 ± 0.9	39.2 ± 3.2	128.9 ± 25.6
*Dura*PETase$^{K95N/S121E/F201I/R280A}$	16.6 ± 13.4	11.3 ± 4.1	13.5 ± 6.9	35.2 ± 6.1
*Dura*PETase$^{K95N/S121E/F201I/N233C/R280A/S282C}$	13.6 ± 5.9	13.9 ± 0.9	22.0 ± 7.5	65.1 ± 8.6

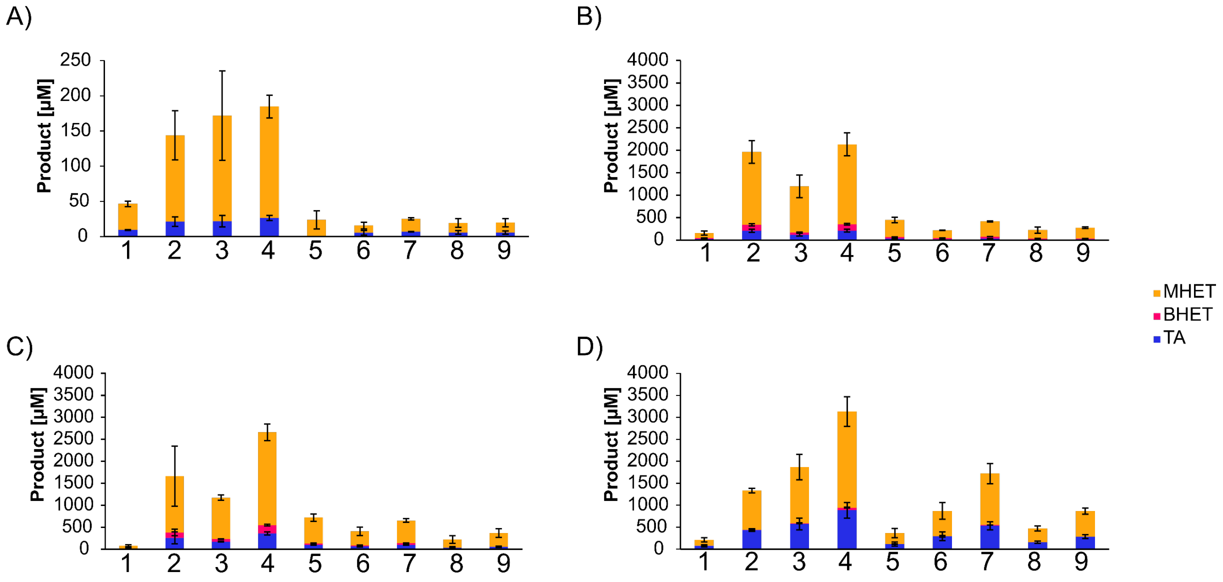

Figure S4: Comparison of degradation products for PET nanoparticles hydrolysis catalyzed by selected *Is*PETase variants after 24 h incubation. Biocatalysis with PET nanoparticles (0.2 mg mL^{-1}) was performed with 30 nM *Is*PETase variant in 50 mM sodium phosphate buffer (pH 7.5) at different incubation temperatures: A) 30°C, B) 40°C, C) 50°C and D) 60°C and a constant agitation of 1000 rpm. The investigated *Is*PETase variants: 1 = *Is*PETaseWT, 2 = *Is*PETaseTM, 3 = *Is*PETaseTM$^{K95N/F201I}$, 4 = *Is*PETaseTM$^{N233C/S282C}$, 5 = *Is*PETaseTM$^{K95N/F201I/N233C/S282C}$, 6 = *Dura*PETase, 7 = *Dura*PETase$^{N233C/S282C}$, 8 = *Dura*PETase$^{K95N/S121E/F201I/R280A}$ and 9 = *Dura*PETase $^{K95N/S121E/F201I/N233C/R280A/S282C}$. The measurement was performed in triplicates and the mean values and standard deviations are given.

Table S4: Relative activities of selected *Is*PETase compared to the *Is*PETase triple mutant for degradation of amorphous PET film at 60°C incubations after 72 h. Instead of the wild type the triple mutant was chosen for comparison because it was the starting point for this study. Biocatalysis was performed with 50 nM *Is*PETase variant in 50 mM glycine-NaOH buffer (pH 9.0) at 60°C incubation and constant agitation of 1000 rpm. Relative activities are based on total product release after 72 h incubation. The mean values and standard deviations are given.

***Is*PETase variant**	**Relative activity [%] compared to *Is*PETaseTM**
*Is*PETaseWT	17.6 ± 3.1
*Is*PETaseTM	100.0 ± 64.3
*Is*PETaseTM$^{K95N/F201I}$	314.3 ±134.7
*Is*PETaseTM$^{N233C/S282C}$	431.6 ± 297.2
*Is*PETaseTM$^{K95N/F201I/N233C/S282C}$	2043.2 ± 854.4
*Dura*PETase	490.5 ± 299.7
*Dura*PETase$^{N233C/S282C}$	1989.5 ± 208.7
*Dura*PETase$^{K95N/S121E/F201I/R280A}$	211.1 ± 52.2
*Dura*PETase$^{K95N/S121E/F201I/N233C/R280A/S282C}$	740.8 ± 398.0

Table S5: Compared melting points [°C] of published *Is*PETase variants.

*Is*PETase variant	This study	Son *et al.* [a]	Zhong-Johnson *et al.* [b]	Cui *et al.* [c]
*Is*PETaseWT	45.1 ± 0.01	48.81	48.1 ± 1.3	46.0
*Is*PETaseTM	56.6 ± 1.55	57.62	-	-
*Is*PETaseTM$^{N233C/S282C}$	68.2 ± 0.03	-	69.4 ± 0.3	-
*Dura*PETase	75.0 ± 0.11	-	-	77.0

a) in 50 mM Na_2HPO_4-HCl buffer (pH 7.0), determined with Applied Biosystems™ protein thermal shift™ dye [2]

b) in 50 mM glycine-NaOH buffer (pH 9.0) supplemented with 50 mM NaCl, determined with differential scanning fluorimetry with Sypro™-Orange dye [5]

c) in 50 mM Na_2HPO_4 buffer (pH 7.5) supplemented with 100 mM NaCl, determined with differential scanning fluorimetry with Sypro™-Orange dye [6]

References

[1] Molecular graphics and analyses performed with UCSF Chimera, developed by the Resource for Biocomputing, Visualization, and Informatics at the University of California, San Francisco, with support from NIH P41-GM103311.

[2] Son, H.F., Cho, I.J., Joo, S., Seo, H., et al., Rational protein engineering of thermo-stable PETase from *Ideonella sakaiensis* for highly efficient PET degradation. *ACS Catal.* 2019, *9*, 3519–3526.

[3] Vogel, K., Wei, R., Pfaff, L., Breite, D., et al., Enzymatic degradation of polyethylene terephthalate nanoplastics analyzed in real time by isothermal titration calorimetry. *Sci. Total Environ.* 2021, *773*:145111.

[4] Pfaff, L., Breite, D., Badenhorst, C.P.S; Bornscheuer, U.T., Wei, R., Fluorimetric high-throughput screening method for polyester hydrolase activity using polyethylene terephthalate nanoparticles, in: Weber, G., Bornscheuer, U.T., Wei, R. (Eds.), Methods Enzymol. Enzymatic Plastic Degradation, Elsevier, 2021, 648, 254-270.

[5] Zhong-Johnson, E.Z.L., Voigt, C.A., Sinskey, A.J., An absorbance method for analysis of enzymatic degradation kinetics of poly(ethylene terephthalate) films. *Sci. Rep.* 2021, *11*:928.

[6] Cui, Y., Chen, Y., Liu, X., Dong, S., et al., Computational redesign of a PETase for plastic biodegradation under ambient condition by the GRAPE strategy. *ACS Catal.* 2021, *11*, 1340–1350.
